# Book Reviews

**Published:** 1904-01

**Authors:** 


### BOOK REVIEWS.

---

A Textbook of Operative Surgery. Covering the Surgical Anatomy and Operative Technic Involved in the Operations of General Surgery. Written for Students and Practitioners. By Warren Stone Bickham, Phar. M., M. D., Assistant Instructor in Operative Surgery, College of Physicians and Surgeons, New York. Octavo, 984 pages, 559 illustrations, entirely original. Philadelphia, New York, London: W. B Saunders & Company. 1903. (Cloth, $6.00 net; sheep or half morocco, $7.00 net.)

To affirm with truthfulness that another treatise on surgery were necessary to the proper understanding of the art might be a somewhat difficult proposition to maintain. On the other hand, one need only glance at some of the features of the one under consideration to determine its usefulness and to be convinced that the art of surgery has been advanced in detail and concrete by its publication.

The author's aim is to lay stress upon surgical anatomy, as related to operative technic and, according to his own foreword, the work is planned to present to the student and practitioner the best technic of modern surgeons in the operations he describes.

He divides his book into two parts, the first dealing with the operations of general surgery, and the second with those of special surgery. He has arranged the consideration of each subject in a very practical manner, giving landmarks, instruments used, description of the operation, preparation and position of the patient, position of the surgeon and his assistants,—these and many other suggestions of importance are presented with each operation. Bickham places great importance very properly upon the incision itself, and gives it a separate heading in each instance. His reason is that the operator who begins an operation correctly has a decided advantage over one who commits an error at the outset.

The table of contents attracts attention from the fact that to each chapter is given a synopsis of its subject-matter,—a plan that will facilitate reference. To give an example, chapter one relates to operations upon arteries, and three quarters of a page is devoted to the synopsis of its contents. If, therefore, one wishes to see a description of the ligation of the lower third of the anterior tibial artery he will find it on page 105. The descriptions of all operations are concise with scarcely a superfluous word, and the author's methods of dealing with almost every subject are original. It must be confessed, however, that sometimes his conciseness is secured at the expense of elegance in diction, even though it has enabled him to compress much valuable material into narrow space. When a description involves the repetition of anatomical topography or other topics, reference is made to the page where such description may be found, instead of rewriting the material.

The latest operations are given with clearness and the best technic for their execution is described. We may cite Bickham's chapters on brain surgery and on the pancreas as instances of excellent detail and as examples of the very best modern scientific surgical instruction. The book abounds with evidence of thoughtful, studious work, is arranged in a manner that appeals to both student and practising surgeon, and will prove of great value to him who must perforce practise surgery as well as medicine, and for which he must equip himself with literature which meets his necessities. Moreover, it will be needed by every teacher and instructor in surgery of whatever grade.

The illustrations are remarkable exhibitions of artistic accuracy. So far as we know, this is the first surgical treatise to put out 559 illustrations that are practically original drawings. Those not entirely so, of which there are but few, have been modified by the author and redrawn under his direction, hence, they too, become substantially original in character. We cannot refrain from bestowing a word of personal praise on the artist who has done all this superb work. Fortunate, indeed, is the author in securing the services of so skilful and conscientious an artist as Miss Fry, who seems to possess a knowledge both of anatomy and surgery, and, therefore, not only knows what she is doing but why she is doing it. Finally, the author has given more complete explanation of each particular picture than usual, which enhances their value more than four-fold.

---

The Practice of Obstetrics. Designed for the use of Students and Practitioners of Medicine. By J. Clifton Edgar, Professor of Obstetrics and Clinical Midwifery in the Cornell University Medical College; Attending Obstetrician to the New York Maternity Hospital. Royal octavo, pages 1111, with 1221 illustrations, many of which are printed in colors. Philadelphia: P. Blakiston's Son & Co. 1903. (Price, cloth $6.00; sheep or half morocco, $7.00.)

In taking up a new treatise on obstetrics for review one can scarcely avoid the mental interrogatory,—"Of what need is there of another?" It must be confessed, too, that this is a pertinent inquiry, since a number of excellent works on obstetrics have appeared within the last few years, for it would seem upon examining them, as if the last word had been said already, for the present at least, on this branch of medical science. But even an imperfect inspection of Edgar's book is sufficient to convince that it contains features of the practice of obstetrics presented in new form, and in such a manner as to attract the attention, as well as fix in the mind the material offered.

The arrangement of the work is such as to appeal alike to the student and to the obstetrician who would refer to it for information in emergencies. There are ten parts placed in the following order: (1) physiology of the female genital organs; (2) physiological pregnancy; (3) pathological pregnancy; (4) physiological labor; (5) pathological labor; (6) physiological puerperium; (7) pathological puerperium; (8) physiology of the newly born; (9) pathology of the newly born; (10) obstetric surgery. This is a logical subdivision of obstetrics, and commends itself even to a tyro, progressing from the simple beginning to the heroic ending, fittingly reserving obstetric surgery until the last. The diagnosis of pregnancy, a subsection of the second part, is a fine piece of instruction and betrays the thoughtful and experienced teacher. Edgar summons all the known methods and arranges them in the order of sequence; and in differential diagnosis it is difficult to see how one could be better armed to meet what is sometimes the most difficult of problems to solve. Again, in the examination of pregnancy, the author has presented much valuable material in the clearest fashion possible, and which will impress the novitiate with the importance of thoroughness. Asepsis is here unfolded and discoursed upon most admirably, the use of rubber gloves being insisted upon as a means to prevent infection.

The description in detail of physiological labor is not excelled in any treatise, and in some respects we think it has never been equaled. This is a topic with which every student should become familiar and he can find no better exposition of it than in this book. Pathological labor is another section in which the author displays great familiarity with his subject and presents it with equally great clearness. Scarcely a complication of labor, even though of the lesser importance, is omitted and the entire field is surveyed with more than ordinary perspicuity. We have never observed a better handling of dystocia in all its details. Edgar divides fever during the puerperium into the septic and nonseptic varieties. It is interesting to note the ingenious methods with which he deals with the last named form of fever. Rarely has this topic received such able treatment.

We have only here and there touched upon some of the excellencies of this work; it must be examined with care to be appreciated and it may be studied by experienced obstetricians with great profit. It is superbly illustrated, most of the pictures being original, some being redrawn modifications of other cuts or plates. Each of the ten principal parts is preceded by a complete synopsis of its contents and the entire construction of the book is beyond criticism. It will take its place among the best books of the year and is second to none as a scientific exposition of the present status of obstetrics.

INFECTIOUS DISEASES. Their Etiology, Diagnosis and Treatment. By G. H. ROGER, Professor Extraordinary in the Faculty of Medicine of Paris, etc., translated by M. S. Gabriel, M. D., New York. In one octavo volume, of 864 pages, with 43 illustrations. Lea Brothers & Co., Philadelphia and New York. 1903. (Cloth, $5.75 net.)

Knowledge of the infections and the diseases they cause is essential to the successful management of the majority of maladies that afflict the human race at the present time. Changes in the methods of practice have steadily followed the advances of the laboratory whenever the researches of the latter have explained the observations of the clinician. To arrive at an adequate understanding of the true meaning of the phenomena of infectious diseases, the laboratory worker and the clinical observer must act in concert; better still, if, as in Professor Roger, the opportunity and the ability for both lines of study are concentrated in one and the same person,—a rare gift.

In this work the rôle of pathogenic bacteria is considered in considerable detail, the microbic associations being an especially interesting chapter, while the defenses and reactions of the organism are developed in an unusual degree. Suppuration and gangrene are given considerable space, while septicemia and pyemia are exhaustively dealt with. After these comes the consideration of the nodular infections, in which is included tuberculosis; then cellular degenerations, and next general reactions in which the phenomena of fever are unfolded. The influence of infections upon the organism furnishes opportunity for extended dissertation of a most profitable character and of a most interesting nature. These four chapters merit the closest examination. Then we come to the evolution of infectious diseases, a chapter that is followed by one on the consequences of infection; these are followed by chapters on the mechanism of immunity and of predisposition, congenital infections and heredity, the diagnosis and prognosis of infectious diseases, the therapeutics of infectious diseases and lastly, the prophylaxis and hygiene of infectious diseases.

From even this cursory view of the general scope and purpose of the work it will be observed that it is of great importance to every practising physician, that it is unlike any other treatise, and that it is a scientific exposition of the status of infectious diseases as understood at the present time. The author is not only perfectly familiar with this subject but he discourses upon it in a most interesting and instructive manner.

---

Physical Diagnosis of Diseases of the Chest. By Richard C. Cabot, M. D., Physician to out-patients, Massachusetts General Hospital. Small octavo, pages 335. One hundred and forty-seven illustrations. Second revised edition. New York: William Wood & Co. 1903. (Price, $2.50.)

The development of knowledge relating to diseases of the chest began with the stethoscope. Austin Flint is quoted everywhere throughout the civilised world, as having contributed much toward the perfection of the test of auscultation and percussion and to the correct interpretation of the sounds elicited thereby. Cabot in the present work, has done much to further advance this method of diagnosis. He has contributed toward the further perfection of our means of examining the chest and of understanding what is heard in that mysterious cavity.

He has written a small treatise that covers the field as admirably, and we might add as completely, as most of the larger works. We have expressed our opinion heretofore (March, 1901, p. 618,) in these columns as to the quality of the material in Cabot's book, and we wish now to reaffirm our previously expressed praise of it. It is a work that will prove of great assistance to the student in his earlier studies and cannot fail to aid the practising physician in his clinical work. The author has made such revisions in this edition as seemed necessary to make it conform to the present state of knowledge relating to diseases of the chest.

A Dictionary of Medical Science. Containing a full explanation of the various subjects and terms of Anatomy, Physiology, Medical Chemistry, Pharmacy, Pharmacology, Therapeutics, Medicine, Hygiene, Dietetics, Bacteriology, Pathology, Surgery, Ophthalmology, Otology, Laryngology, Dermatology, Gynecology, Obstetrics, Pediatrics, Medical Jurisprudence, Dentistry, Veterinary Science, etc. By Robley Dunglison, M. D., LL. D., late Professor of Institutes of Medicine and Medical Jurisprudence in the Jefferson Medical College of Philadelphia. New (twenty-third) edition, thoroughly revised, with the pronunciation, accentuation and derivation of the terms, by Thomas L. Stedman, A. M., M. D., Fellow of the New York Academy of Medicine. Imperial octavo, 1224 pages, 600 illustrations, with thumb-letter index. Philadelphia and New York: Lea Brothers & Co. 1903. (Cloth, $8.00 net; leather, $9.00 net; half morocco, $9.50 net.)

The first edition of this dictionary was issued in 1839, and it has continued to occupy a prominent place in the libraries of every well-read physician since that time. Through frequent editions it has kept pace with changes and additions in the medical vocabulary until as now presented it is as near complete as any similar work of its size and scope. After the death of the learned author, the work was carried through several editions by his distinguished son, Dr. R. J. Dunglison, who numbered among other accomplishments that of being a lexicographer of the first order. Upon his death, the publishers were compelled to choose another editor, and they have been fortunate in securing the services of Dr. Stedman in that capacity. He has done good service in the revision under consideration, and has made it a word-book that will take the front rank among medical dictionaries. It is of equal value to undergraduate and practitioner, to student of science and to literateurs everywhere, and in it is evidenced the fact that, no matter what progress is made in any department of medical science, Dunglison's medical dictionary is destined to keep pace with it and to continue in the future, as in the past, to be one of the best exponents of medical improvement.

---

The Standard Medical Directory of North America. 1903-4. Including a Directory of Practising Physicians in the United States of America, Canada, Cuba, Mexico and Central America. Lists of Practitioners of the Specialties and Alphabetical Index of all Physicians in North America. Also Directories of Medical Officers of the United States Army and Navy, Medical Societies, Medical Colleges, Medical Laws and Boards, Medical Publications (Books and Periodicals), Hospitals and Sanitariums, Mineral Springs, Drugs and Medicines, Medical and Surgical Products, Manufacturers and Life Insurance Companies. Chicago: G. P. Engelhard & Co. (Price, $10.00.)

The title above given embraces an excellent synopsis of the contents of this valuable directory. First of all, it is an address-book of the physicians of the North American continent; then it is a guide to hospitals, sanitariums, colleges of medicine, medical societies, instrument makers, and book publishers; also it gives the medical laws of the several states, territories and countries. Its list of medical books and periodicals is a novel and useful part of the work. It is the first catalogue of medical publications that we have seen which approaches anywhere near perfection, and it can be consulted with confidence in its accuracy.

The book is well bound in red buckram, which is a point of importance. A poorly bound book is inconvenient to examine and soon becomes useless, especially one consulted so frequently as a directory. It is surprising that so much information can be collected from so many sources with so few errors, and we apprehend a great demand for this excellent work.

INTERNATIONAL CLINICS. A Quarterly of Illustrated Clinical Lectures and especially prepared original articles on Treatment, Medicine, Surgery, Neurology, Pediatrics, Obstetrics, Gynecology, Orthopedics, Pathology, Dermatology, Ophthalmology, Otology, Rhinology, Laryngology, Hygiene and other topics of interest to students and practitioners, by leading members of the medical profession throughout the world. Edited by A. O. J. KELLY, A.M., M.D., Philadelphia. Volume III. Thirteenth series. 1903 Philadelphia: J. B. Lippincott Company. 1903. (Cloth, $2.00.)

The contents of this volume of the clinics include diseases of the gall-bladder and gall-ducts, six articles; treatment, four articles; medicine, four articles; and surgery, six articles. Among the American contributors may be found the names of Musser, Stockton, Deaver, Mays and Belfield. Among the foreign contributors are such men as Finlay, Fowler, Murray, Championniére, Robin, Rudolph and Schwartz. This number is not as profusely illustrated as some of the previous volumes, but it contains much material of great interest. It forms a component of this great series that is a credit to all concerned in creating it.

THE PHYSICIANS' VISITING LIST (Lindsay and Blakiston's) for 1904. Philadelphia: P. Blakiston's Son & Company, 1012 Walnut Street. Sold by all booksellers and druggists.

It is needful in these days of business hurry and worry for every physician to be supplied with those conveniences which economise his time to the best advantage. One of the most important time-savers is a good visiting list. Lindsay and Blakiston's adequately fills all the necessities of such a convenience. Its size is such as to fit the pocket nicely; it is well made of thin, fine paper, with gilt edges, bound in leather, has a pocket, a rubber-tipped pencil, and contains numerous memoranda relating to clinical facts, such as doses of drugs, antidotes for poisons, obstetrical table and the like. The prices range from 75 cents to $2.25, according to the size of the book required. This is the fifty-third successive year of its publication which is a sufficient justification of all the praise that may be said of it.

---

THE MEDICAL RECORD VISITING LIST OR PHYSICIANS' DIARY FOR 1904. New revised edition. New York: William Wood & Company.

One of the most popular visiting lists is that published by William Wood & Company and known as the Medical Record Visiting List. It contains a group of miscellaneous facts useful at the bedside, such as dosage, emergency measures, obstetric calendar and blank pages for a variety of records, in addition to the ordinary pages allotted to the daily visitation of patients. It can be had at prices ranging from $1.25 to $4.00 according to size, style and quality of binding, the average adapted to the needs of most practitioners being arranged for 30 and 60 patients a week and selling at $1.25 and $1.50. Orders should be placed promptly to avoid delay in delivery.

